# Nanoparticle Decorated Ultrathin Porous Nanosheets as Hierarchical Co_3_O_4_ Nanostructures for Lithium Ion Battery Anode Materials

**DOI:** 10.1038/srep20592

**Published:** 2016-02-05

**Authors:** Jawayria Mujtaba, Hongyu Sun, Guoyong Huang, Kristian Mølhave, Yanguo Liu, Yanyan Zhao, Xun Wang, Shengming Xu, Jing Zhu

**Affiliations:** 1 Beijing National Center for Electron Microscopy, School of Materials Science and Engineering, The State Key Laboratory of New Ceramics and Fine Processing, Key Laboratory of Advanced Materials (MOE), Tsinghua University, Beijing 100084, China; 2 Department of Micro- and Nanotechnology, Technical University of Denmark, 2800 Kongens Lyngby, Denmark; 3 Institute of Nuclear and New Energy Technology, Tsinghua University, Beijing 100084, China; 4 School of Metallurgy and Environment, Central South University, Changsha 410083, China; 5 School of Resources and Materials, Northeastern University at Qinhuangdao, Qinhuangdao 066004, China; 6 Department of Chemistry, Tsinghua University, Beijing 100084, China

## Abstract

We report a facile synthesis of a novel cobalt oxide (Co_3_O_4_) hierarchical nanostructure, in which crystalline core-amorphous shell Co_3_O_4_ nanoparticles with a bimodal size distribution are uniformly dispersed on ultrathin Co_3_O_4_ nanosheets. When tested as anode materials for lithium ion batteries, the as-prepared Co_3_O_4_ hierarchical electrodes delivered high lithium storage properties comparing to the other Co_3_O_4_ nanostructures, including a high reversible capacity of 1053.1 mAhg^−1^ after 50 cycles at a current density of 0.2 C (1 C = 890 mAg^−1^), good cycling stability and rate capability.

Rechargeable lithium-ion batteries (LIBs) are one of the great successes of modern materials electrochemistryused in electronics, mobile phones, and laptop computers^1^,^2^. High-performance LIBs with higher capacity, longer cycle life, and better rate capability have attracted considerable interests in the electric vehicle market and for implantable medical devices etc^1^,^2^,^3^,^4^,^5^. It is widely accepted that the overall performance of LIBs is highly dependent on the inherent electrochemical properties of the electrode materials^6^,^7^. Therefore, considerable attention has been paid to develop novel materials for both the cathodes and anodes of LIBs which are inexpensive, safe and environmentally benign. So far, various materials, such as graphitic/non-graphitic carbon^8^,^9^,^10^, transition-metal oxides^11^,^12^, nitrides^13^,^14^, phosphates^15^, lithium alloys^16^,^17^,^18^,^19^,^20^ and their composites have been exploited as the anode materials of LIBs. Among them, Co_3_O_4_ is a promising material for the LIB anode due to its high theoretical capacity (890 mAhg^−1^, according to the electrochemical reaction Co_3_O_4_ + 8Li^+^ + 8e^−^ ↔ 3Co + 4Li_2_O). The capacity is more than two times larger than that of graphite (372 mAhg^−1^), which is anticipated to meet the requirements of future energy storage systems^21^. Nevertheless, the main weakness of Co_3_O_4_ anode materials for LIBs lies in the large volume expansion and severe particle aggregation associated with the Li^+^ insertion and extraction process, resulting in the deterioration of the reversible capacity and poor cycling stability^21^. Various strategies have been attempted to overcome these limitations and improve the performance. Design and synthesis of electrode materials with proper composition, morphology (such as nanopowders, nanowires, nanorods and nanotubes), and microstructure on the nanoscale is a time-tested route to enhance the lithium storage properties^22^,^23^,^24^,^25^ (see Co_3_O_4_ anode nanostructures overview in Table S1). Li *et al*.^24^ syntheized self-supported mesoporous Co_3_O_4_ nanowire arrays directly grown on Ti foil, which showed high capacity, good cyclability and high rate capability. Nam and Belcher *et al*.^25^ used viruses to synthesize and assemble Au modified Co_3_O_4_ nanowires at room temperature, and demostrated the improved battery capacity when used as anodes for LIBs. Combining nanostructured electrode materials with electronically conductive agents, such as carbon nanofibers, carbon nanotubes, and graphene, is considered as another effective approach to improve the cycling stability and rate capability^26^,^27^. The conductive additives not only act as a “buffer zone” of volume variation induced by the cycling process but also a good electron transfer medium^28^,^29^,^30^,^31^. However, the addition of conductive agents unavoidably decreases the effective use of active materials.

Recent works show that three-dimensional (3D) complex hierarchical architectures assembled by low-dimensional nano-sized building blocks possess enhanced LIB performance^32^,^33^,^34^. For example, Sun and co-workers^32^ developed a high-performance cathode material based on nickel-rich lithium transition-metal oxide with full concentration gradient within each particle, and the micrometre-size secondary particles of the material were composed of aligned needle-like nanosize building blocks. The developed material could deliver a specific capacity of up to 215mAhg^−1^ with outstanding cycling stability in a full-cell configuration, maintaining 90% capacity retention after 1,000 cycles. We previously reported a facile synthesis of 3D hierarchical porous Co_3_O_4_ nanostructures with morphologies including hierarchical nanoflowers and hyperbranched nanobundles, which were all built up by numerous nanoparticles with random attachment. Of those, the nanoflowers demonstrated the highest performance as an anode materials for LIBs^35^.

Herein, we report a novel material based on crystalline core-amorphous shell nanoparticles decorating ultrathin nanosheets of hierarchical Co_3_O_4_ nanostructures (Co_3_O_4_ C@A NPs-NSs HNs). In this structure, crystalline@amorphous core/shell Co_3_O_4_ nanoparticles with a bimodal size distribution were decorated on ultrathin Co_3_O_4_ nanosheets. When evaluated as an anode material for LIBs, the Co_3_O_4_ C@A NPs-NSs HNs delivered a reversible capacity of 1053.1 mAhg^−1^ after 50 cycles at a current density of 0.2 C (1 C = 890 mAg^−1^), good cycling stability and rate capability. The lithium storage properties, especially the rate capacity of the current Co_3_O_4_ C@A NPs-NSs HNs were superior to the reported Co_3_O_4_ nanostructures. The improved performance was ascribed to the unique designed nanostructured Co_3_O_4_.

## Results

The route to obtain Co_3_O_4_ C@A NPs-NSs HNs was adapted from our previously described synthesis of mesoporous Co_3_O_4_ nanosheets with some modifications (see the Supporting Information (SI) for details). Briefly, the cobalt hydroxide sheet precursors were obtained by reacting Co(NO_3_)_2_·6H_2_O, hexamethylenetetramine, and water at 95 °C for 8 h. The product was then annealed at 450 °C in air for 2 h to form Co_3_O_4_ nanosheets that have previously been investigated^36^. In the last step, the annealed samples were soaked in NaBH_4_ solution for an hour to yield C@A NPs-NSs HNs.

The morphology of the as prepared precursors shows sheet-like character (Fig. S1a, Supporting Information). EDX pattern confirms the composition of the precursor (Fig. S1a inset, SI). Powder X-ray diffraction (XRD) result indicates all the diffraction peaks can be indexed as Co(OH)_2_ (JCPDS No. 74–1057) (Fig. S1b, SI). The thermal behavior of the Co(OH)_2_ precursor is studied by TG analysis (Fig. S2, SI). From the TGA curve measured under air atmosphere, it can be clearly seen that the weight loss takes place in the temperature range 50–220 °C, and it is mainly attributed to the removal of physically adsorbed water and partial decomposition of the solid precursor into Co_3_O_4_ nanosheets. After reaching 250 °C, the weight loss drops sharply to about 30%. Then the weight loss is gradual and finally it flattens at 350 °C. On the basis of these results the as-synthesized precursors were annealed at 450 °C. The structure and morphology characterizations indicate that the annealed products are phase pure Co_3_O_4_ with mesoporous nanosheets (Co_3_O_4_ mNSs) nature (Figs S3 and S4, SI).

**Figure 1:**
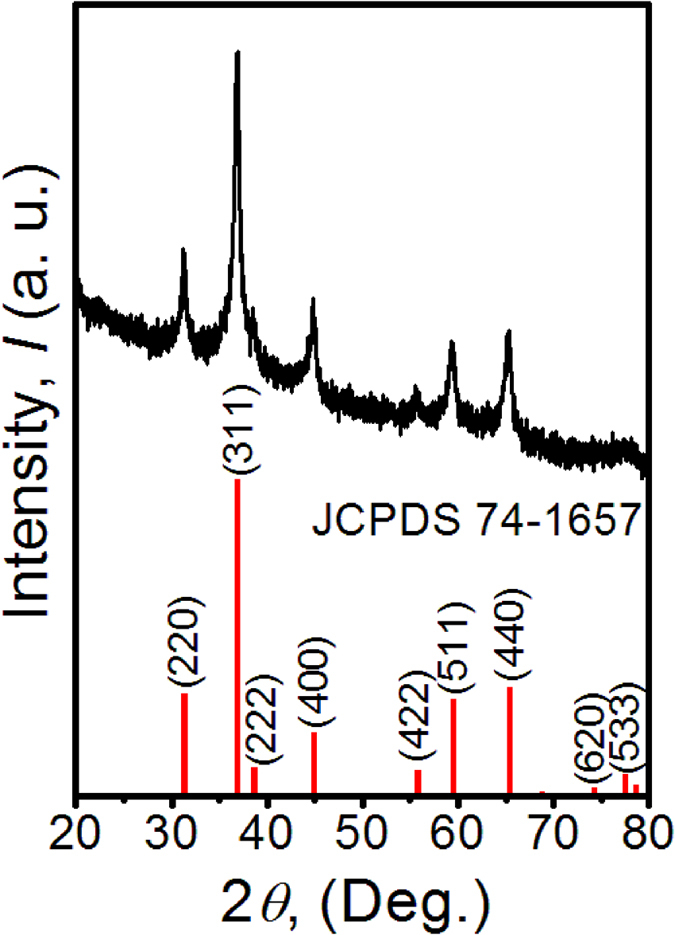
XRD pattern of as-prepared sample and the standard pattern of Co_3_O_4_ phase.

The final Co_3_O_4_ C@A NPs-NSs HNs can be obtained by soaking the annealed Co_3_O_4_ mNSs in NaBH_4_ solution for an hour. The crystallographic structure of Co_3_O_4_ C@A NPs-NSs HNs are checked by XRD as shown in Fig. 1. All the diffraction peaks can be assigned to (220), (311), (222), (400), (422), (511), (440), (620) and (533) planes of Co_3_O_4_ (JCPDS No. 74–1657, a = 8.0837 Å). No other diffraction peaks from possible impurities are observed, indicating the high phase purity of the Co_3_O_4_ C@A NPs-NSs HNs. The diffraction peaks for Co_3_O_4_ C@A NPs-NSs HNs are not as sharp as that of starting Co_3_O_4_ mNSs, implying the crystallinity of Co_3_O_4_ C@A NPs-NSs HNs is reduced after soaking in NaBH_4_ solution.

**Figure 2:**
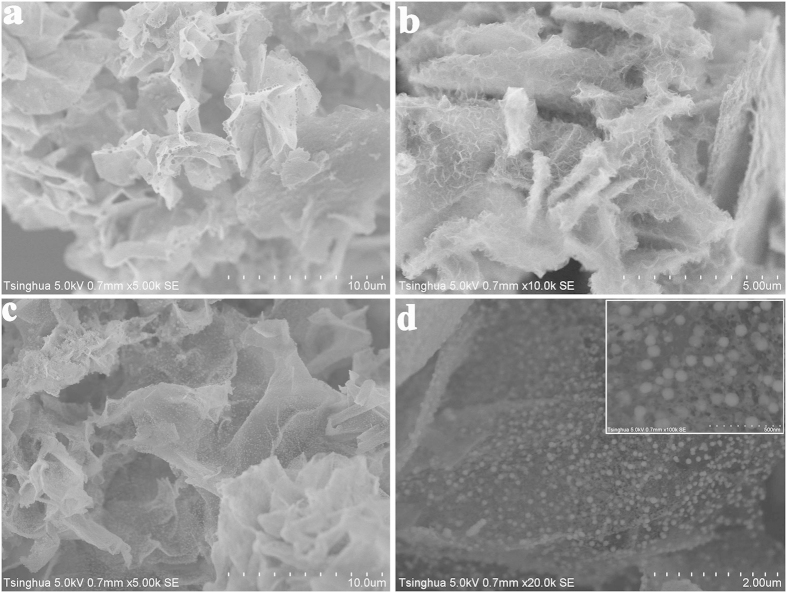
FESEM images of the Co_3_O_4_ nanohybrids with different magnifications.

The morphology of Co_3_O_4_ C@A NPs-NSs HNs is examined by field emission scanning electron microscopy (FESEM) as shown in Fig. 2. It can be seen that the surface of Co_3_O_4_ C@A NPs-NSs HNs is coarse and shows wrinkled morphology (Fig. 2a,b), in sharp contrast with that for Co_3_O_4_ mNSs (Fig. S4a, SI). An enlarged FESEM image (Fig. 2c) clearly shows that numerous Co_3_O_4_ nanoparticles are uniformly distributed on the surface of the nanosheets. A higher magnification FESEM image shows that two kinds of Co_3_O_4_ nanoparticles (small size and large size) are observed on the surface (Fig. 2d and the inset). The average diameters of the two kinds of nanoparticles are ~6 and ~45 nm, respectively. Although various 0D nanoparticles (metal, metal oxides/sulfide/nitrides) have been successfully deposited on 2D nanosheets (such as graphene, reduced graphene oxide, layered transition metal dichalcogenide nanosheets, atomically-thick nanosheets with non-layered structure, and so on)^27^,^37^,^38^,^39^, to the best of our knowledge, this is the first report on the synthesis of 0D-2D hybrid nanostructures for a given material via a facile solution method.

**Figure 3:**
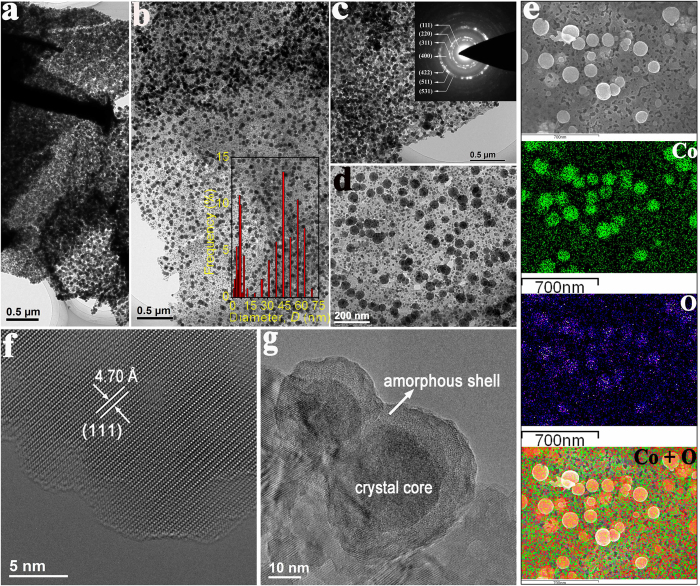
(**a–d**) TEM images of Co_3_O_4_ C@A NPs-NSs HNs, with the insets in (**b**,**c**) showing the size distribution profile of Co_3_O_4_ nanoparticles and SAED pattern. (**e**) is SEM elemental mapping image showing the homogenous distribution of elements of Co and O in Co_3_O_4_ C@A NPs-NSs HNs. (**f**,**g**) are HRTEM images of nanosheet and core/shell nanoparticles, respectively.

The detailed structural investigations of Co_3_O_4_ C@A NPs-NSs HNs are studied by TEM and HRTEM. Figure 3(a–d) are typical TEM images of Co_3_O_4_ C@A NPs-NSs HNs with different magnifications; it can be seen that the hierarchical nanosheets are highly porous (Fig. S5a,b). The sheets are folded in some locations and the dark regions are the result of overlapping of sheets. Moreover, the porous nanosheets are decorated with high-density nanoparticles. A bimodal size distribution with mean diameters of ~5.5 nm and ~43 nm are obtained by analyzing over 200 nanoparticles for the sample (the inset in Fig. 3b). The results are in good agreement with the above FESEM observations. A selected area electron diffraction pattern (Fig. 3c inset) clearly demonstrates the polycrystalline nature of Co_3_O_4_ C@A NPs-NSs HNs, which is consistent with the XRD results.

EDS mapping analysis indicates the uniform distribution of cobalt and oxygen in the product (Fig. 3e), confirming the composition of supported Co_3_O_4_ nanoparticles as well. The HRTEM image of an individual Co_3_O_4_ nanosheet from the hierarchical nanostructures is shown in Fig. 3(f). The distinct lattice spacing is measured to be ~4.70 Å, which corresponds to the (111) plane of Co_3_O_4_. HRTEM image shown in Fig. 3(g) illustrates that the Co_3_O_4_ nanoparticles are supported on the nanosheets. The nanoparticles possess unique crystalline core-amorphous shell structure. Additional HRTEM images for the nanoparticles are shown in Fig. S5, further demonstrating the crystalline@amorphous core/shell nature of Co_3_O_4_ nanoparticles.

**Figure 4:**
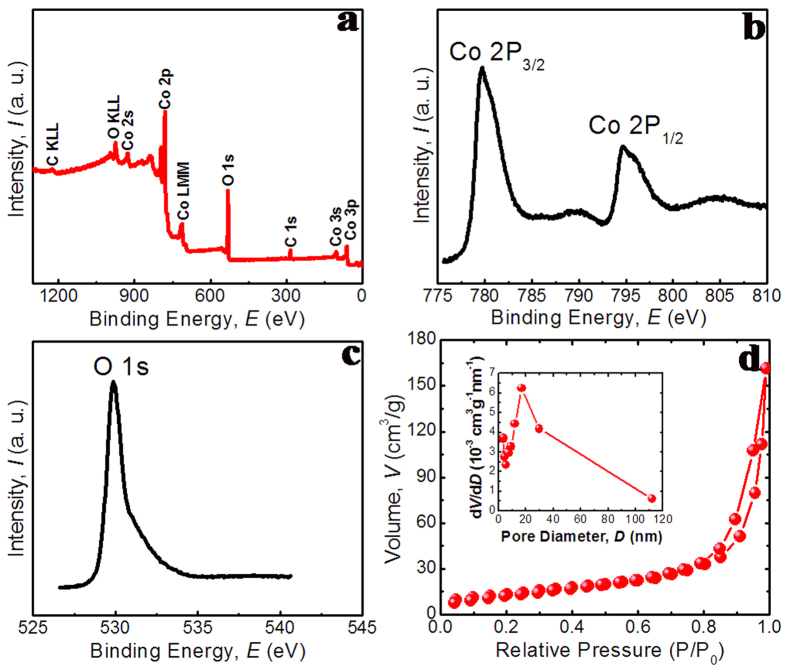
(**a**) XPS survey spectra of Co_3_O_4_ nanohybrids, (**b,c**) high-resolution XPS spectra of the Co 2p and O 1s regions, respectively, (**d**) Nitrogen adsorption–desorption isotherms and corresponding pore size distribution curve (inset) of the Co_3_O_4_ nanohybrids.

The surface chemical composition and oxidation state of Co_3_O_4_ C@A NPs-NSs HNs are determined by employing XPS analysis. The XPS spectrum of the HNs in the region of 0–1300 eV is shown in Fig. 4(a) and confirms the peaks of Co and O. The high resolution scan of Co 2p (Fig. 4b) exhibits two peaks located at 795.6 eV and 780.2 corresponding to the electronic states of Co 2p_1/2_ and Co 2p_3/2_ respectively. The presence of Co_3_O_4_ can be further confirmed by the O 1s peak (Fig. 4c) located at 530.2 eV, which corresponds to the oxygen species forming oxide with cobalt elements.

Nitrogen adsorption–desorption isotherm is used to determine the specific surface area and the porous nature of the product. The N_2_ adsorption-desorption isotherm of Co_3_O_4_ C@A NPs-NSs HNs at 77K is presented in Fig. 4(d) with the inset displaying the corresponding Barret–Joyner–Halenda (BJH) pore size distribution. The isotherm shows a hysteresis loop at relative pressure range of 0.8–1.0 P/P_0_ and the Bruauer–Emmett–Teller (BET) specific surface area is 51.9 m^2^g^−1^, which is higher than that of Co_3_O_4_ nanosheets^36^. The higher surface area of Co_3_O_4_ C@A NPs-NSs HNs may be attributed to the mesoporous nature of the nanosheets and the void spaces between the nanoparticles. The BJH pore size distribution curves indicate that the average pore size is ~16 nm for large pores and ~2 nm for small pores (inset in Fig. 4d). The present porous Co_3_O_4_ C@A NPs-NSs HNs are of importance in lithium-storage process, due to their capability of providing extra active sites for the storage of lithium ions and facilitating mass diffusion and ion transport, which are induced by the synergistic reactions of porous structures and the specially designed structure of the constituent nanoparticles.

**Figure 5:**
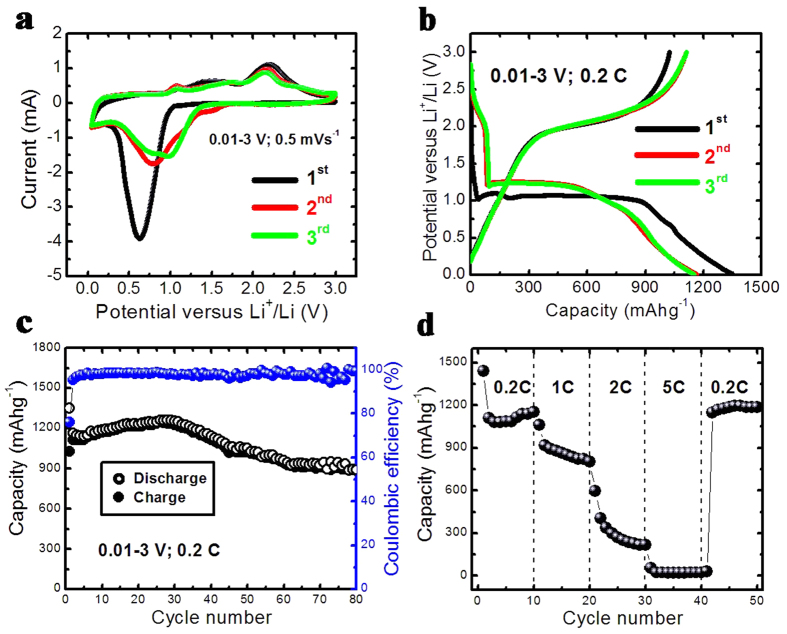
Electrochemical measurements of Co_3_O_4_ nanohybrids: (**a**) CVs at a scan rate of 0.5 mV s^−1^ between 0.01 and 3 V, (**b**) galvanostatic charge/discharge voltage profiles for the 1st, 2nd, and 3rd cycles between 0.01 and 3 V versus Li/Li^+^ at a current density of 0.2 C, (**c**) cycling performance at a constant current rate of 0.2 C between 0.01 and 3 V and (**d**) rate capability at various current rates between 0.2 C and 5 C.

We subsequently study the electrochemical properties of Co_3_O_4_ C@A NPs-NSs HNs as an anode material for LIBs. Figure 5(a) displays the representative cyclic voltammograms (CVs) for the first three cycles at a scan rate of 0.5 mV s^−1^ in the voltage window between 0.01 and 3 V (versus Li^+^/Li). In the first cycle, there is a dominant cathodic peak at ~0.62 V which can be ascribed to the electrochemical reduction (lithiation) reaction of Co_3_O_4_ with Li. In the following anodic scan the anodic peak at ~2.2 V is ascribed to the oxidation (delithiation) reaction of Co_3_O_4_ C@A NPs-NSs HNs. In the second cycle, the main reduction and oxidation peaks are shifted to ~0.78 V and ~2.16 V respectively, and the intensity of reduction peaks decreases due to the formation of irreversible solid electrolyte interface (SEI) film and the irreversible reduction reaction^40^,^41^. The peaks intensity and the integral areas of the third cycle are very close to that of the second cycle. These results show a gradual improvement in the electrochemical reversibility of Co_3_O_4_ C@A NPs-NSs HNs after the first cycle.

Figure 5b shows the representative galvanostatic charge-discharge voltage profiles of Co_3_O_4_ C@A NPs-NSs HNs at a current density of 0.2 C (1 C = 890 mAhg^−1^) in the voltage window of 0.01–3 V (versus Li^+^/Li) at room temperature. Similar to the previous report^40^,^41^, in the first discharge curve, the potential value quickly falls to the plateau (~1.00 V), and the extended plateau with a ~900 mAh/g capacity may likely be ascribed to the conversion from Co_3_O_4_ to Co, and then gradually declines to the cutoff voltage (0.01 V), which could be associated with the formation of a polymer/gel-like film on the surface of Co_3_O_4_ particles^40^,^41^. The electrode delivers first-cycle discharge and charge capacities of 1349.4 and 1025.6 mAhg^−1^, respectively, yielding an irreversible capacity loss of 24%. Such initial irreversible capacity loss mainly originates from the formation of SEI layer due to the irreversible degradation of the electrolyte and other irreversible side reactions^22^,^23^,^24^,^25^,^26^,^35^,^40^. The following two (2^nd^ and 3^rd^) discharge/charge curves tend to be stable and exhibit similar electrochemical behavior. The discharge and charge capacities are 1165.3 and 1111.8 mAhg^−1^ for the second cycle, and 1150.2 and 1112.2 mAhg^−1^ for the third cycle, corresponding to the Coulombic efficiency of 95.4% and 96.7%, respectively. We notice that all of the capacities as mentioned above are higher than the theoretical total capacity of Co_3_O_4_ (890 mAhg^−1^), which is probably caused by the reversible formation/dissolution of the polymer/gel-like film contributing to an additional reversible capacity besides the electrochemical conversion reaction between cobalt oxide and Co^40^,^41^. Figure 5(c) shows the discharge-charge cycling performance of Co_3_O_4_ C@A NPs-NSs HNs evaluated between 0.01 and 3V (versus Li^+^/Li) at a current density of 0.2 C at room temperature. It is found that the capacities of the following cycles from the fourth cycle increase slowly and gradually. The maximum discharge capacities could reach up to 1262 mAhg^−1^. The possible reason is that the diffusion of Li-ion is activated and stabilized gradually during cycling process^42^. The capacities then fall slowly and nearly keep constant after 80 cycles. A reversible capacity of 888.8 mAhg^−1^ can be retained after 80 cycles.

To further evaluate the rate capability, the Co_3_O_4_ C@A NPs-NSs HNs electrode is cycled at various current densities between 0.2 C and 5 C as shown in Fig. 5(d). The charge/discharge rates are programmably modified from 0.2 C to 1 C, 2 C, 5 C and then back to 0.2 C for 10 cycles. It can be found that the discharge and charge capacities remain stable and decrease regularly with an increased current rate. After every 10 cycles at a specific current rate, the reversible capacity at 0.2, 1, 2, and 5 C are about 1151.9, 804, 216.9 and 22.2 mAhg^−1^, respectively. When the current density is decreased from 5 to 1 C, the reversible capacity can be recovered to 1186.3 mAhg^−1^. These results demonstrate that the Co_3_O_4_ C@A NPs-NSs HNs electrode has good electrochemical reversibility.

## Discussion

The lithium-storage properties, including the capacity, cycling performance especially the rate capacity of the Co_3_O_4_ C@A NPs-NSs HNs electrode are superior to that of most nanostructured Co_3_O_4_ materials, such as porous Co_3_O_4_ NWs arrays, Co_3_O_4_ nanowires, flower-like porous Co_3_O_4_ spheres, porous Co_3_O_4_ needles, and so on (see Table S1). The good lithium storage properties of Co_3_O_4_ C@A NPs-NSs HNs might be attributed to the rationally designed hierarchical nanostructures. Firstly, the porous nanosheets and the amorphous shell of the Co_3_O_4_ nanoparticles can accommodate the local volume change upon charge/discharge cycling and is likely to alleviate the problem of pulverization and aggregation of the electrode material, thus leading to improved cycling stability^43^,^44^,^45^,^46^,^47^. Furthermore, the hierarchical architecture assembled with 0D nanoparticles and 2D nanosheets is favorable for preventing the aggregation of the constituted nanobuilding blocks, also improving the cycling performance^35^,^48^. Secondly, the hierarchical structures with high density Co_3_O_4_ decorated nanosheets provide sufficient electrode-electrolyte contact area for the storage of lithium ions, which is beneficial for enhancing the specific capacity. Moreover, the amorphous shell could offer additional reaction sites on the surface, which is also responsible for the high specific capacity of the Co_3_O_4_ C@A NPs-NSs HNs electrodes^43^,^45^,^47^. Finally, the crystalline Co_3_O_4_ cores and nanosheets have the functions of providing stable mechanical support and an efficient electrical conducting pathway, while the amorphous Co_3_O_4_ shells could give reliable continuous pathways for Li^+^ during the course of continuous charge/discharge processes, enhancing the kinetics and structural stability for lithium storage^47^. As a result of the above-mentioned advantages, the prepared Co_3_O_4_ C@A NPs-NSs HNs electrode expectedly manifests enhanced lithium storage properties.

In conclusion, we report the synthesis of unique Co_3_O_4_ C@A NPs-NSs HNs, in which crystalline@amorphous core/shell Co_3_O_4_ nanoparticles with a bimodal size distribution uniformly disperse on ultrathin Co_3_O_4_ nanosheets. When used as the anode materials of LIBs, the as-prepared Co_3_O_4_ C@A NPs-NSs HNs electrodes delivered high lithium storage properties, including a high reversible capacity of 1053.1 mAhg^−1^ after 50 cycles at a current density of 0.2 C, good cycling stability and rate capability. It is believed that the excellent electrochemical performance can be attributed to the uniquely designed hierarchical nanostructures. The present facile synthesis route can be applied to other metal oxides with desirable nanostructures and functions.

## Methods

### Materials Synthesis

All of the reagents are analytical grade and used as received. Firstly, 1.45 g of Co(NO_3_)_2_·6H_2_O and 1.4 g of HMT (hexamethylenetetramine, C_6_H_12_N_4_) were dissolved in 30 ml of water under stirring for 30 min. The mixture was then transferred into a Teflon-linked stainless steel autoclave (50 ml capacity). The autoclave was sealed and maintained at 95 °C for 8 h. After cooling down to room temperature spontaneously, the precipitate is rinsed with distilled water and ethanol, and dried at 60 °C under vacuum for 2 h. Finally,the product was annealed at 450 °C in air for 2 h. The annealed samples were then soaked in 1M NaBH_4_ solution (20 ml distilled water). The sample was collected after an hour and washed with distilled water three times. Finally, the product was collected after centrifugation and dried in an oven at 70 °C for 10 h.

### Characterization of Materials

The phases of the unreduced and reduced products were characterized by X-ray diffraction (XRD). The morphology and structure of the products were obtained by field emission scanning electron microscopy (FESEM, Hitachi S5500), transmission electron microscopy (TEM, FEI Tecnai G^2^ 20, 200 kV), high resolution transmission electron microscopy (HRTEM, FEI Titan 80–300, 300 kV), and X-ray photoelectron spectroscopy (XPS, Escalab 250, Al Kα). The elemental mapping was done by using energy dispersive X-ray spectroscopy (EDS). The surface area of the products was measured by the Bruauer–Emmett–Teller (BET) method using nitrogen adsorption–desorption isotherm. Pore size distribution plots were obtained by the Barret–Joyner–Halenda (BJH) method. Thermogravimetric (TG) analysis was carried out on a TGA 2050 thermogravimeteric analyzer under an air atmosphere at the temperature range of 25–500 °C with a heating rate of 10 °C min^−1^.

### Electrochemical Measurements

To measure the electrochemical performance, the electrodes were constructed by mixing the active materials, conductive carbon black and carboxymethyl cellulose, in a weight ratio of 80:10:10. The mixture was prepared as slurry and spread onto copper foil. The electrode was dried under vacuum at 120 °C for 5 h to remove the solvent before pressing. Then the electrodes were cut into disks (12 mm in diameter) and dried at 100^o^C for 24 h in vacuum. The cells were assembled inside an Ar-filled glove box by using a lithium metal foil as the counter electrode and the reference electrode and microporous polypropylene as the separator. The electrolyte used was 1M LiPF_6_ dissolved in a mixture of ethylene carbonate (EC), propylene carbonate (PC), and diethyl carbonate (DEC) with a volume ratio of EC/PC/DEC = 3:1:1. The assembled cells were allowed to soak overnight, and then the electrochemical tests on a LAND battery testing unit were performed. Galvanostatic charging and discharging of the assembled cells were performed at different current rates between voltage limits of 0.01 and 3V (vs. Li^+^/Li) at room temperature. The cyclic voltammogram (CV) was performed at a scan rate of 0.5 mVs^−1^ in the range of 0.01−3.00 V (vs. Li^+^/Li).

## Additional Information

**How to cite this article**: Mujtaba, J. *et al*. Nanoparticle Decorated Ultrathin Porous Nanosheets as Hierarchical Co_3_O_4_ Nanostructures for Lithium Ion Battery Anode Materials. *Sci. Rep.* **6**, 20592; doi: 10.1038/srep20592 (2016).

## Supplementary Material

Supplementary Information
