# Supplementary material for: Nanoparticle Decorated Ultrathin Porous Nanosheets as Hierarchical Co3O4 Nanostructures for Lithium Ion Battery Anode Materials
Source: Sci Rep. 2016 Feb 5;6:20592. doi: 10.1038/srep20592 (PMC4742879; doi:10.1038/srep20592)
Supplement: Supplementary Information [file srep20592-s1.doc]

**Supplementary Information for**

**Nanoparticle Decorated Ultrathin Porous Nanosheets as Hierarchical Co_3_O_4_ Nanostructures for Lithium Ion Battery Anode Materials**

Jawayria Mujtaba ^1^, Hongyu Sun ^1,2^, Guoyong Huang ^3,4^, Kristian Mølhave ^2^, Yanguo Liu ^5^, Yanyan Zhao ^1^, Xun Wang ^6^,

Shengming Xu ^3^, Jing Zhu ^1^

^1^Beijing National Center for Electron Microscopy, School of Materials Science and Engineering, The State Key Laboratory of New Ceramics and Fine Processing, Key Laboratory of Advanced Materials (MOE), Tsinghua University, Beijing 100084, China, ^2^ Department of Micro- and Nanotechnology, Technical University of Denmark, 2800 Kongens Lyngby, Denmark, ^3^Institute of Nuclear and New Energy Technology, Tsinghua University, Beijing 100084, China, ^4^School of Metallurgy and Environment, Central South University, Changsha 410083, China, ^5^School of Resources and Materials, Northeastern University at Qinhuangdao, Qinhuangdao 066004, China, ^6^Department of Chemistry, Tsinghua University, Beijing 100084, China

Correspondence and requests for materials should be addressed to H. S or Z. J. e-mail: [hysuny@mail.tsinghua.edu.cn](mailto:hysuny@mail.tsinghua.edu.cn); [jzhu@mail.tsinghua.edu.cn](mailto:jzhu@mail.tsinghua.edu.cn)

**Supplementary Figures:**


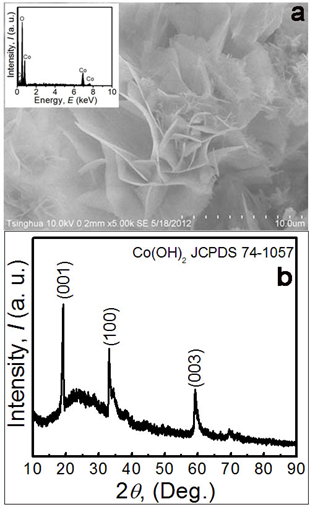


**Figure S1.** (a) Typical SEM image and EDX pattern (inset) of Co(OH)_2_ precursor. (b) XRD pattern of the pure Co(OH)_2_ precursor.


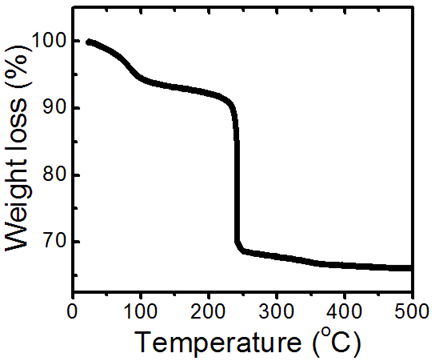


**Figure S2.** TGA curve of Co(OH)_2_ precursor measured at the temperature range of 25–500°C with a heating rate of 10°C under air atmosphere.


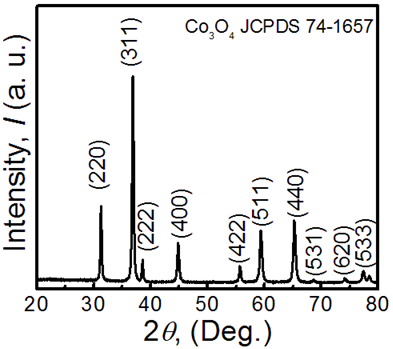


**Figure S3.** XRD spectrum of the pure Co_3_O_4_ nanosheets.


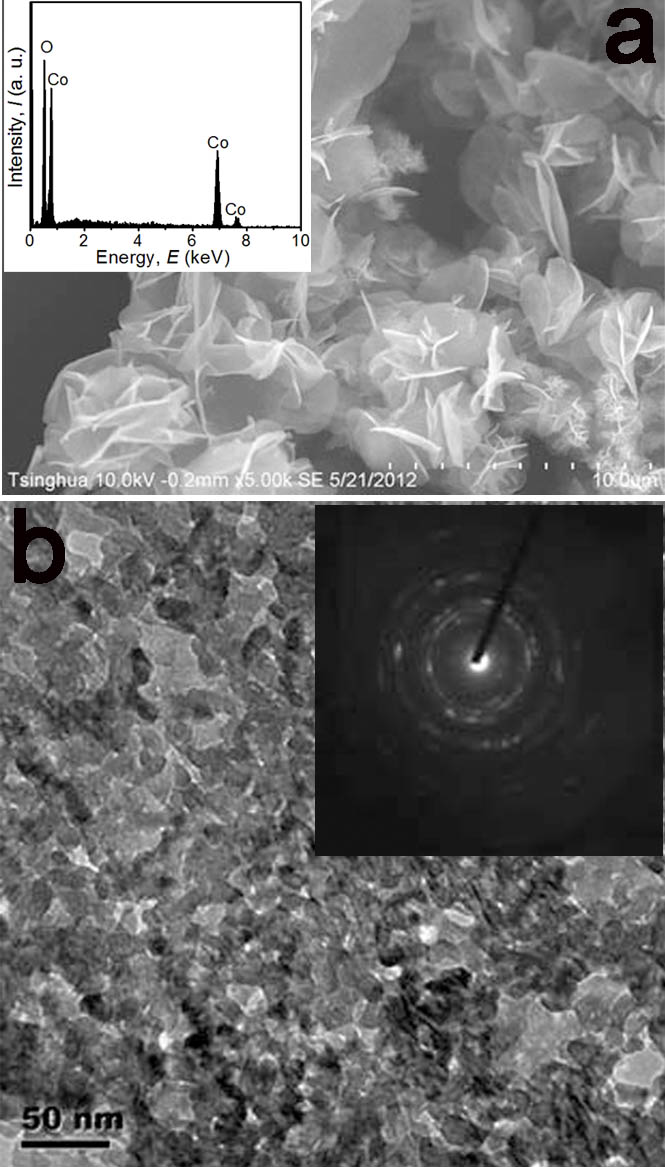


**Figure S4.** (a) Typical SEM image and EDX pattern (inset) of pure Co_3_O_4_ nanosheets. (b) TEM image of pure Co_3_O_4_ nanosheets. The inset shows the corresponding SAED pattern.


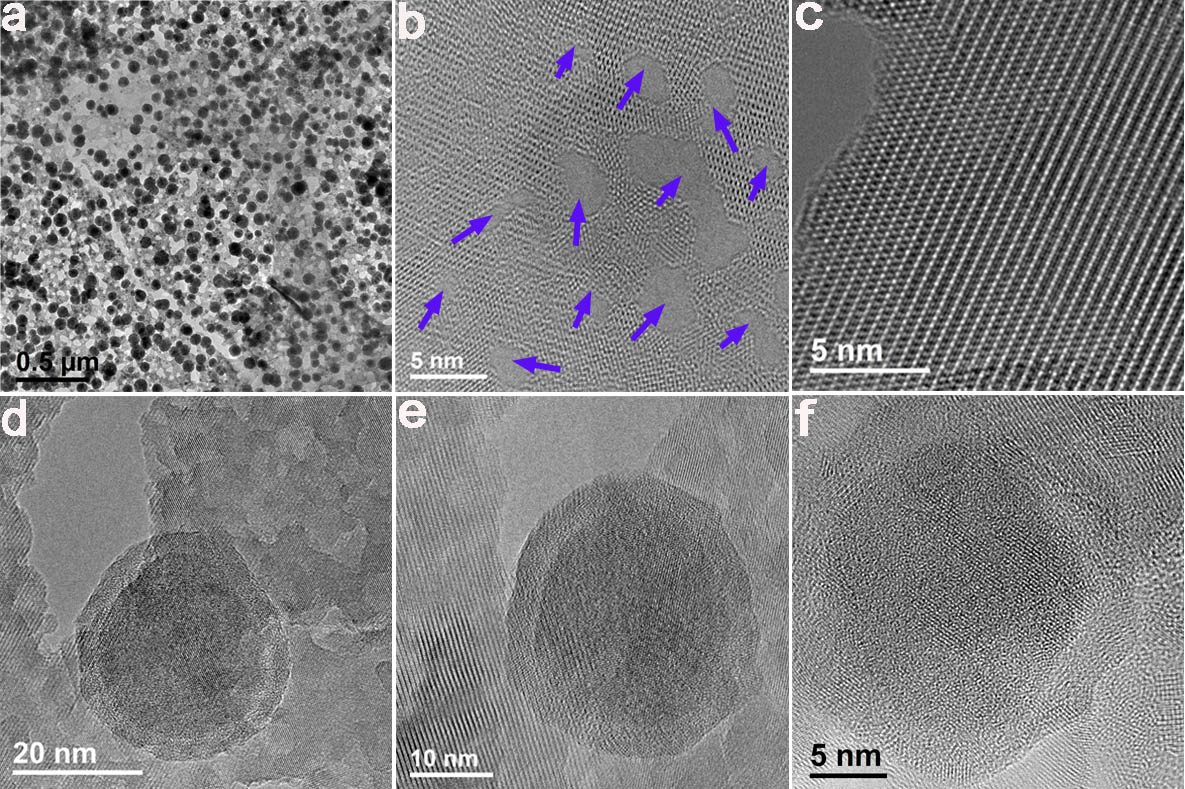


**Figure S5.** Additional TEM and HRTEM images of Co_3_O_4_ C@A NPs-NSs HNs.

**Table S1.** Performance comparison of some LIB anode materials based on different Co_3_O_4_ nanostructures

|  | **Current density**  **/mA g^-1^** | **1^st^ discharge/charge capacity**  **/mAh g^-1^** | **Capacity fading after cycles /mAh g^-1^** | **Ref** |
| --- | --- | --- | --- | --- |
| Porous Co_3_O_4_ NWs arrays | 50 | 1280/1000 | 1520 | 1 |
| Co_3_O_4_ NWs | 110 | 1050/700 | 680 | 2 |
| Mesoporous Co_3_O_4_ NWs arrays | 50 | 1732/1100 | 1220 | 3 |
| Flower-like porous Co_3_O_4_ spheres | 50 | 1316.7/970 | —— | 4 |
| Porous Co_3_O_4_ needles | 445 (0.5 C) | 1542/1100 | —— | 5 |
| Macroporous Co_3_O_4_ platelets | 100 | 1380/970 | 970 | 6 |
| porous Co_3_O_4_ nanoflowers | 50 | 1849/1196 | 980 | 7 |
| Co_3_O_4_ nanobundles | 50 | 1564/980 | 600 | 7 |
| Co_3_O_4_ nanoparticles | 50 | 1105/817 | 300 | 8 |
| Co_3_O_4_ C@A NPs-NSs HNs | 178 (0.2 C) | 1349.4/1025.6 | 888.8 | 9 |

1C = 890 mAg^-1^

1. Wang, J. *et al*. Microwave homoge-neous synthesis of porous nanowire Co_3_O_4_ arrays with high capacity and rate capability for lithium ion batteries. *Mater. Chem. Phys*. 126, 747 (2011).
2. Yao, X. *et al*. Co_3_O_4_ nanowires as high capacity anode materials for lithium ion batteries. *J. Alloys Compd*. 521 (2012) 95.
3. Xiong, S. *et al*. Mesoporous Co_3_O_4_ and CoO@C transformed from chrysanthemum-like Co(CO_3_)_0.5_(OH)_0.11_H_2_O and their lithium-ion battery application. *Adv. Funct. Mater*. **22**, 861 (2011).
4. Zheng, J. *et al*. A Facile synthesis of flower-like Co_3_O_4_ porous spheres for the lithium-ion battery electrode. *J. Solid State Chem*. **183**, 600 (2010).
5. Xue, X. Y. *et al*. Porous Co_3_O_4_ nanoneedle arrays growing directly on copper foils and their ultrafast charg-ing/discharging as lithium-ion battery anodes. *Chem. Commun*. **47**, 4718 (2011).
6. Lu, Y. *et al*. Macroporous Co_3_O_4_ platelets with excellent rate capability as anodes for lithium ion batteries. *Electrochem. Commun*. **12**, 101 (2010).
7. Sun, H. *et al*. Morphology-controlled synthesis of Co_3_O_4_ porous nanostructures for the application as lithium-ion battery electrode. *Electrochim. Acta* **89**, 199 (2013).
8. Wu, Z. S. *et al*. Graphene anchored with Co_3_O_4_ nanoparticles as anode of lithium ion batteries with enhanced reversible capacity and cyclic performance. *ACS Nano* **4**, 3187 (2010).
9. This work.
